# The Glacier Ice Worm, *Mesenchytraeus solifugus*, Elevates Mitochondrial Inorganic Polyphosphate (PolyP) Levels in Response to Stress

**DOI:** 10.3390/biology11121771

**Published:** 2022-12-06

**Authors:** Teresa Osorio, Ernest R. Scoma, Daniel H. Shain, Diana S. Melissaratos, Lindsey M. Riggs, Vedangi Hambardikar, Maria E. Solesio

**Affiliations:** Department of Biology and CCIB, College of Arts and Sciences, Rutgers University, 201 Broadway, Camden, NJ 08103, USA

**Keywords:** inorganic polyphosphate, polyP, ATP, annelids, stress, ice worms, *M. solifugus*, bioenergetics

## Abstract

**Simple Summary:**

Energy maintenance in living organisms is crucial for survival. The ice-obligate worm, *Mesenchytraeus solifugus*, displays an unusual bioenergetic pattern, namely that intracellular ATP levels increase with declining temperature. In this study, we address the effects of stress on mitochondrial inorganic polyphosphate (polyP) and its relationship with ATP. Mitochondrial inorganic polyphosphate is a ubiquitous polymer whose role in the maintenance of prokaryotic and mammalian bioenergetics has been broadly demonstrated. We show here that polyP levels in ice worms increase with thermal stress, in contrast with those observed in other annelid worms. Thus, polyP may function as an energetic buffer in ice worms, effectively storing phosphate groups under stress and replenishing ATP under normal physiological conditions.

**Abstract:**

The inorganic polymer, polyphosphate (polyP), is present in all organisms examined to date with putative functions ranging from the maintenance of bioenergetics to stress resilience and protein homeostasis. Bioenergetics in the glacier-obligate, segmented worm, *Mesenchytraeus solifugus*, is characterized by a paradoxical increase in intracellular ATP levels as temperatures decline. We show here that steady-state, mitochondrial polyP levels vary among species of Annelida, but were elevated only in *M. solifugus* in response to thermal stress. In contrast, polyP levels decreased with temperature in the mesophilic worm, *Enchytraeus crypticus*. These results identify fundamentally different bioenergetic strategies between closely related annelid worms, and suggest that I worm mitochondria maintain ATP and polyP in a dynamic equilibrium.

## 1. Introduction

Primary energy generation pathways are well conserved across domains of life, and bioenergetic regulation is fundamental to the survival of all organisms [1]. Inorganic polyphosphate (polyP)—a negatively charged polymer associated with bioenergetics—comprises monomers of orthophosphate linked by high-energy phosphoanhydride bonds, which are isoenergetic to those present in ATP [2]. A rapid inter-conversion of phosphoryl groups between ATP and polyP has been observed in yeast [3]. Accordingly, the regulatory role of polyP in cellular bioenergetics has been broadly demonstrated [4,5,6,7,8,9,10,11,12,13,14,15,16,17] For example, polyP facilitates the enzymatic synthesis of ADP in *Escherichia coli* [13] and mediates primary metabolism in yeast [11]. In mammalian cells, we and others have demonstrated the functional role of mitochondrial polyP in (i) the regulation of mitochondrial free calcium crucial for ATP production [18], (ii) preserving the equilibrium between oxidative phosphorylation (OXPHOS) and glycolysis [15,16], and (iii) maintenance of the appropriate oxidative status of cells [17,19]. 

Mitochondrial inorganic polyphosphate is present in all studied organisms, from prokaryotes to mammals [20], showing a mostly ubiquitous cellular distribution. In eukaryotes, polyP can be found in various subcellular locations including the cytoplasm, nucleus and mitochondria, and can be associated with membrane proteins as well as the extracellular space [8]. In mammalian cells, a co-localization between polyP and mitochondria has been shown [5,12]. Moreover, polyP levels are highly dynamic and closely coupled with the metabolic state of mitochondria [15,16,17,21]. Recent evidence suggests that the mitochondrial F_0_F_1_ ATP synthase is linked with the metabolism of mammalian polyP [22]. 

Glacier ice worms, *Mesenchytraeus solifugus*, display an atypical bioenergetic property, namely that ATP levels paradoxically increase with declining temperatures [23]. This has been interpreted as a compensatory mechanism to offset reductions in molecular motion and enzyme kinetics at cold physiological temperatures [23,24]. Ice worms complete their life cycle in hydrated, maritime glacier ice found throughout the Pacific northwestern region of North America [25,26], and thus are challenged with permanently cold temperatures hovering near 0 °C. To explore the putative role of polyP in the maintenance of mitochondrial bioenergetics in these worms, we tested their response to thermal stress and hypoxia in comparisons with mesophilic, aquatic (*Helobdella austinensis* and *Lumbriculus variegatus*) and terrestrial (*Eiseniella andrei* and *Enchytraeus crypticus*) worms. Our data show that steady-state mitochondrial polyP levels vary significantly between worms occurring at different habitats, and that *M. solifugus* uniquely elevates polyP as a function of increasing temperature. 

## 2. Material and Methods

### 2.1. Reagents

FCCP (Carbonyl cyanide 4-(trifluoromethoxy) phenylhydrazone), Tris-HCl, NaCl, EDTA, SDS (sodium dodecyl sulfate), sucrose, mannitol, PMSF (phenylmethylsulfonyl fluoride), protease and phosphatase inhibitors, and TMRM (tetramethylrhodamine methyl ester, perchlorate) were purchased from Sigma-Aldrich (St. Louis, MO, USA). Dulbecco’s Modified Eagle medium (DMEM), heat-inactivated fetal bovine serum (FBS), penicillin-streptomycin, trypsin, DAPI (4′-6-diamino-2-phenylindole) and Proteinase K were purchased from ThermoFisher Scientific (Waltham MA, USA). PolyP standards were a gift from Dr. Toshikazu Shiba, from Kitasato University, Tokyo, Japan. 

### 2.2. Specimens

Glacier ice worms, *Mesenchytraeus solifugus*, were collected from surface snow above the equilibrium line altitude on The South Sister Glacier (OR, USA). Specimens were stored in thermally insulated containers with field snow/ice during transport to Rutgers University, upon which they were transferred into glass bowls and maintained at 4 °C. No additional water or supplements were added to ice worm cultures. Specimens of *Lumbriculus variegatus* were purchased from local pet stores and maintained in 0.03% Instant Ocean (Blacksburg, VA, USA) equilibrated to 19 °C in glass bowls. Terrestrial worms, *Enchytraues crypticus*, were maintained in a laboratory colony with rich topsoil supplemented weekly with oatmeal and water, housed in covered plastic containers. Red wiggler earthworms, *Eiseniella andrei*, were purchased locally and identified by PCR barcoding. Worms were maintained in the absence of food (e.g., ice worms are maintained for over two years without feeding [23]), except *E. crypticus*, which was equilibrated to experimental temperatures for at least 24 h without food prior to harvesting; thus, any contribution of gut contents to the analyses was minimized.

### 2.3. Polymerase Chain Reaction (PCR) Species Identification

DNA extractions were performed with a Qiagen Blood and Tissue kit (Germantown, MD, USA), according to the manufacturer’s specifications. For PCR, 1 μL of template was added to 24 μL of premixed DreamTaq (FisherScientific, Waltham, MA, USA) solution supplemented with 4 μM each of cytochrome *c* oxidase subunit 1 (CO1) universal primers HCO and LCO [27]. Cycling conditions were 95 °C–2 min; 95 °C–20 s; 50 °C–40 s; 72 °C–40 s; 35X. Reactions were screened on a 1% agarose gel viewed under UV light and positives were run through a GenJet PCR Clean-up kit (SignaGen, Frederick, MD, USA). Sanger sequencing was performed by Azenta (South Plainfield, NJ, USA) on both strands using HCO and LCO primers, respectively.

### 2.4. Dry Weight Quantification

Samples were weighed using a laboratory analytical balance and incubated in a Lindberg Blue M oven (FisherScientific, Waltham, MA, USA), at 55 °C for 24 h. Weights were recorded again after 55 °C incubation to obtain wet:dry weight ratios. 

### 2.5. Mitochondrial Isolation

Mitochondrial isolation was adapted from current protocols [4,17,28]. Specifically, samples were mechanically minced with a razor blade and lysed in a solution containing 50 mM Tris-HCl (pH 7.4), 100 mM NaCl, 1 mM EDTA, 0.5% SDS and 2 mg/mL proteinase K. Samples were incubated at 50 °C for 60 min under agitation (300 rpm). Subsequently, samples were incubated at 80 °C for 5 min to inactivate proteinase K, and mitochondria were isolated as described [4,17,28]. Protein concentration was quantified as described previously [29].

### 2.6. PolyP Quantification

Samples were lysed as described above for mitochondrial isolation. PolyP-DAPI shifts the fluorescence emission towards higher wavelengths (λ_excitation_ = 405 nM), compared to wavelengths needed to visualize DNA-DAPI [30]. Utilizing this property, standard curves with synthetic polyP (0–10 μM) were employed to quantitate mitochondrial polyP. Briefly, 5 μL of isolated mitochondria was loaded in triplicate into 96-well black plates (transparent bottom) and incubated in the presence of 20 µM DAPI. After 15 min in the dark and at room temperature, plates were read using a BioTek spectrophotometer (ThermoFisher Scientific) as described [15,17]. Mitochondrial inorganic polyphosphate concentrations were normalized to wet:dry weight ratios. 

### 2.7. Temperature-Induced Stress

Specimens were equilibrated and incubated at stated temperatures for 24 h under aquatic or terrestrial conditions, accordingly. Temperatures were chosen based on worms’ viable thermal range.

### 2.8. Hypoxia

Specimens were placed in 6-well plates in a hypoxia chamber (COY, Grass Lake, MI, USA) under 0.1% O_2_ atmosphere at their preferred temperature for 24 h. Subsequently, samples were lysed, and DAPI-PolyP assays were conducted as described above.

### 2.9. Mitochondrial Membrane Potential Assay

Freshly prepared mitochondria were isolated from worm specimens as described above. Protein quantitation was performed using a nanodrop spectrophotometer (Nanodrop2000, ThermoFisher Scientific). Subsequently, 40 μg of protein was loaded into 96-well plates and incubated in the presence of 200 nM TMRM for 20 min at 37 °C. After incubation, plates were read using a BioTek spectrophotometer (ThermoFisher Scientific). Lastly, 20 μM FCCP was loaded into each well, the samples were left for 10 min at room temperature, and fluorescence was measured again, using the same parameters.

### 2.10. Statistical Analysis

Experiments and analyses were conducted in triplicate. In each case, one independent worm was used per experiment. Triplicates were conducted with different worms. Data are expressed as the mean ± SEM. Statistical analyses were carried out using Origin Lab (Northampton, MA, USA). Data were analyzed using non-parametric tests. Specifically, Chi-squared test was used in all the figures comparing more than two groups of data and Mann–Whitney U test was used in remaining Figures. Values of *p* ≤ 0.05 were considered significant (* *p* ≤ 0.05, ** *p* ≤ 0.01, *** *p* ≤ 0.001). 

## 3. Results

**Figure 1:**
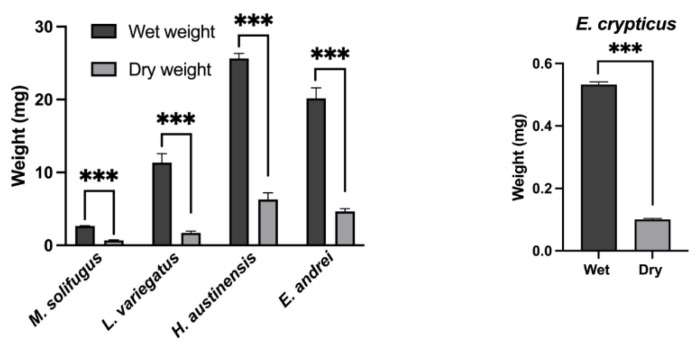
**Annelida included in this study have different wet and dry weight values.** Wet and dry weight values were calculated for all the animals used on this study. The levels of polyP were normalized considering these values. Data are expressed as mean ± SEM of, at least, three independent experiments conducted using biological triplicates. Values of *p* ≤ 0.05 were considered significant (*** *p* ≤ 0.001).

**Figure 2:**
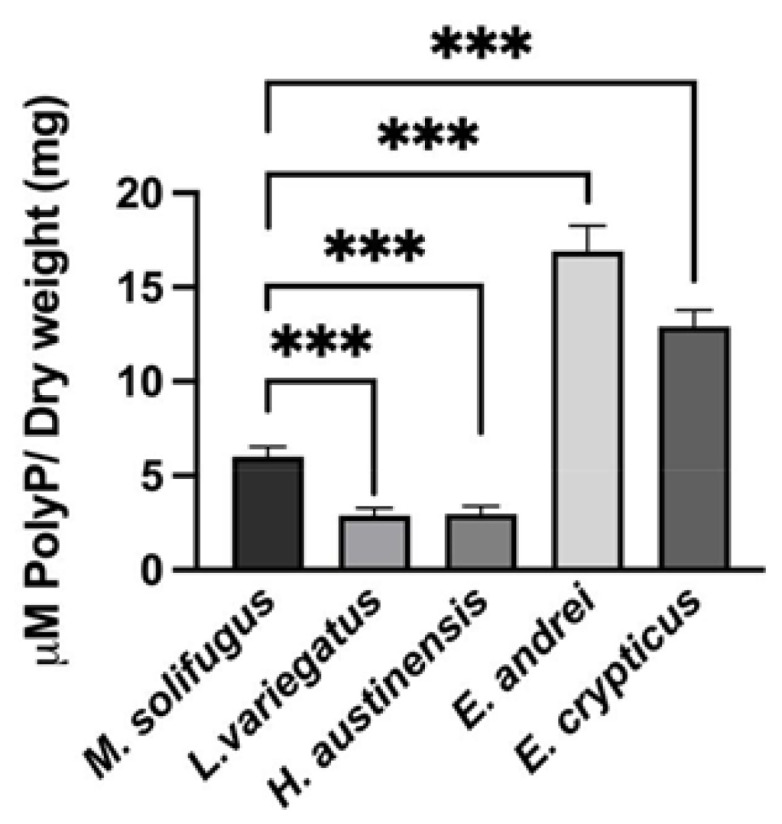
**Annelida show different levels of mitochondrial polyP under basal conditions.** The levels of polyP within mitochondria were measured under control conditions. Our results show that all the mesophilic counterparts had different levels of polyP within mitochondria. In fact, ice worms had intermediate concentrations of polyP, when compared with the other Annelida that were studied. Data are expressed as mean ± SEM of, at least, three independent experiments, conducted using biological triplicates. Values of *p* ≤ 0.05 were considered significant (*** *p* ≤ 0.001).

**Figure 3:**
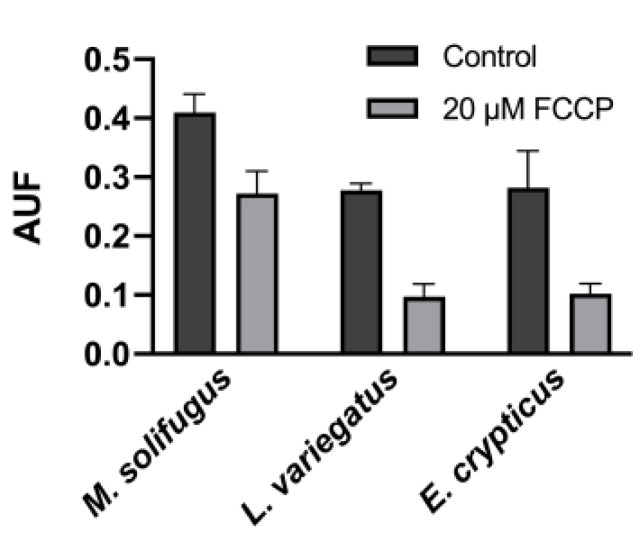
**The differences in the levels of mitochondrial polyP observed in the annelids are not a consequence of a lack of mitochondrial membrane potential.** All Annelida used to conduct this study had mitochondrial membrane potential, assayed by TMRM fluorescence. Data are expressed as mean ± SEM of, at least, three independent experiments, conducted using biological triplicates. Values of *p* ≤ 0.05 were considered significant.

**Figure 4:**
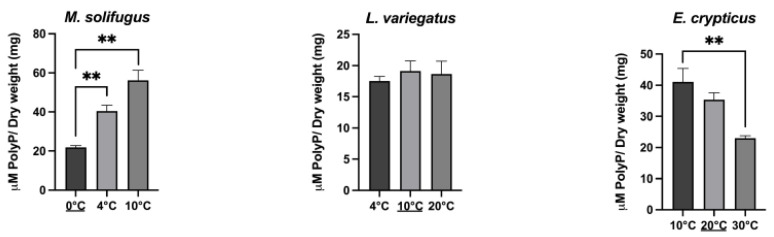
**Increased temperature increases the levels of mitochondrial polyP in ice worms, but not in the other mesophilic counterparts.** Using the same methods to assay polyP as under control conditions, we observed a significant temperature-dependent increase in the levels of polyP in *M. solifugus,* which was not present in the other mesophilic counterparts. Underlined temperature is the optimal temperature for each of the annelids. Data are expressed as mean ± SEM of, at least, three independent experiments conducted using biological triplicates. Values of *p* ≤ 0.05 were considered significant (** *p* ≤ 0.01).

**Figure 5:**
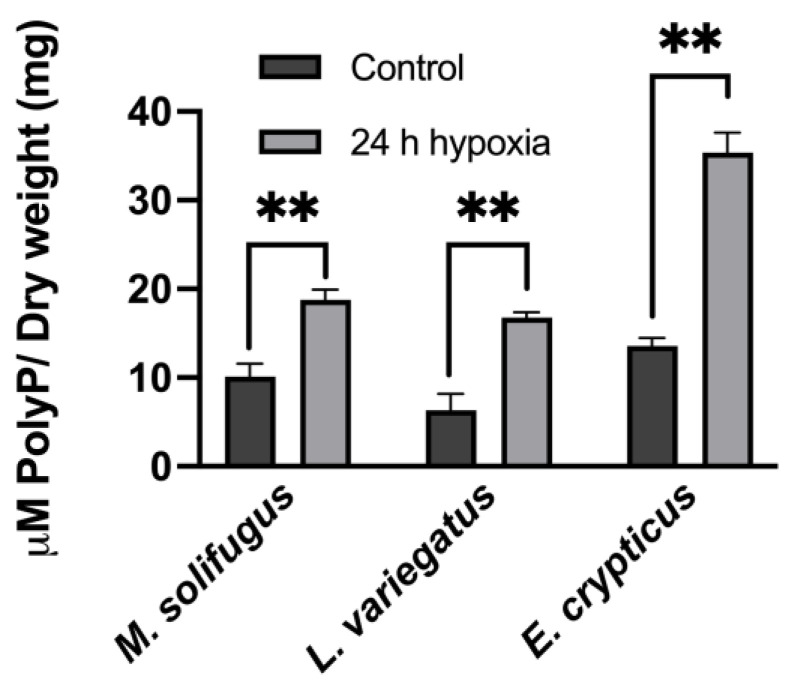
**When stress is induced by hypoxia, all Annelida included in our study showed a rise in the levels of polyP.** The effects of hypoxia in the levels of mitochondrial polyP were also assayed in our samples. However, no major differences were found in *M. solifugus* and the other mesophilic counterparts. Data are expressed as mean ± SEM of, at least, three independent experiments conducted using biological triplicates. Values of *p* ≤ 0.05 were considered significant (** *p* ≤ 0.01).

To establish steady-state levels of mitochondrial polyP across a diverse group of annelids, we assayed DAPI-polyP fluorescence using specific wavelengths, as described [15]. Mitochondrial inorganic polyphosphate levels were monitored in mitochondria isolated from five species of annelid worms: *Mesenchytraeus solifugus* (glacial), *Lumbriculus variegatus* (aquatic), *Helobdella austinensis* (aquatic), *Eiseniella andrei* (terrestrial) and *Enchytraeus crypticus* (terrestrial). Since water content differed between worms, wet and dry weights were calculated and normalized for all comparisons (Figure 1). Our data show that polyP levels were significantly different between worms occurring at different habitats, with highest levels observed in terrestrial species (*E. andrei* and *H. austinensis*) and lowest in aquatic species (*L. variegatus* and *H. austinensis*) (Figure 2). This prompted us to assay mitochondrial membrane potential (using TMRM fluorescence) among worm species to confirm that mitochondria were functionally equivalent; comparable values were observed in all worms examined, but decoupling appeared to be less efficient in *M. solifugus* (Figure 3). Subsequently, we measured mitochondrial polyP levels as a function of temperature within each worm’s viable thermal range (i.e., deviating accordingly from its preferred temperature). Mitochondrial polyP levels increased significantly with temperature in *M. solifugus*, remained relatively constant in *L. variegatus* and decreased significantly in *E. crypticus* (Figure 4). Since oxygen solubility decreases with temperature, we measured polyP levels in response to hypoxia (also an independent stressor), a condition in which all worms significantly increased polyP (Figure 5). 

## 4. Discussion

Our results show that disparate annelid worms display highly variable mitochondrial polyP levels under basal conditions. Considering that wet:dry ratios were relatively constant between these worms, it remains unclear why polyP levels were significantly elevated in terrestrial species. Nonetheless, these differences are suggestive of bioenergetic patterns that distinguish these species, particularly the glacier ice worm, *Mesenchytraeus solifugus*. For example, *Lumbriculus variegatus* (aquatic) and *Enchytraeus crypticus* (terrestrial) displayed similar membrane potential:decoupling ratios, but these appeared to be reduced in *M. solifugus*, suggesting an enhanced ability of the latter to depolarize the mitochondrial membrane (see Figure 3). Importantly, temperature is a well-known modulator of protein dyshomeostasis [31], and possibly the stenothermic environment of ice worms (i.e., ~0 °C over geological time) stabilizes machinery associated with membrane potential; alternatively, the apparent requirement for elevated ATP in *M. solifugus* [23,24] may have provided strong evolutionary pressure to select for robust OXPHOS coupling in these worms.

Under thermal stress, levels of mitochondrial polyP increased significantly in *M. solifugus*, in contrast to their mesophilic counterparts, where levels were either not altered (*L. variegatus*) or decreased (*E. crypticus*). These opposite responses have been observed elsewhere; specifically, bacterial polyP increases with stress [32,33], while mammalian polyP levels could decrease with stress. In fact, levels of mitochondrial polyP are dependent on the status of mitochondrial physiology, and they decrease as mitochondria becomes dysfunctional [21]. Mitochondrial dysfunction has been broadly described under stress conditions [34]. These differences seem to reflect fundamentally different strategies for maintaining bioenergetic activity in the context of polyP. In bacteria and in vitro systems, polyP has been proposed to function as a molecular chaperone [35,36,37]. Since protein chaperoning is a highly energy-dependent process [38], the roles of polyP as a regulator of bioenergetics and of protein homeostasis could be linked. In mammalian cells, polyP is thought to regulate levels of mitochondrial free calcium, as well as opening the mitochondrial permeability transition pore [4,5,14]. Both are closely related to the generation of ATP via OXPHOS. The depletion of mitochondrial polyP in these organisms has a deleterious effect on OXPHOS function, entirely disrupting cellular bioenergetics [15,16,17].

The functional role of polyP as a molecule of energy storage has been demonstrated by us and others [21,39]. Thus, a kinetically favorable interconversion between the two highly energetic molecules, ATP and polyP, could buffer their behavior and place them in a dynamic equilibrium. The unique response of *M. solifugus* to thermal stress, namely an increase in mitochondrial polyP as a function of temperature (see Figure 4), is in direct contrast with a paradoxical decrease in ATP observed in these worms [23,24]. Taken together, it appears that mitochondrial polyP in *M. solifugus* is positioned to donate high energy phosphates to ADP in an energy deficit cellular environment, similar to a phosphagen [40], and may otherwise function to stabilize proteins critical to maintaining mitochondrial function (e.g., membrane potential), in a chaperone-like role [35,41,42].

## 5. Conclusions

Our data suggest that polyP and ATP are in a dynamic equilibrium within ice worm mitochondria, such that the paradoxical decline of ATP with temperature is coupled with a corresponding increase in polyP. These observations are consistent with a putative role of polyP as a stress-related chaperone, similar to what has been proposed in bacteria. Importantly, this response appears to be ice worm-specific amongst Annelida based on our analyses of disparate segmented worms, suggesting a novel bioenergetic function within this worm lineage.

## Data Availability

Further information and requests for resources and reagents should be directed to and will be fulfilled by the corresponding author.

## References

[1] Wallace D.C. (2010). Colloquium paper: Bioenergetics, the origins of complexity, and the ascent of man. Proc. Natl. Acad. Sci. USA.

[2] Morrissey J.H., Choi S.H., Smith S.A. (2012). Polyphosphate: An ancient molecule that links platelets, coagulation, and inflammation. Blood.

[3] Saiardi A. (2012). How inositol pyrophosphates control cellular phosphate homeostasis?. Adv. Biol. Regul..

[4] Solesio M.E., Garcia Del Molino L.C., Elustondo P.A., Diao C., Chang J.C., Pavlov E.V. (2020). Inorganic polyphosphate is required for sustained free mitochondrial calcium elevation, following calcium uptake. Cell Calcium..

[5] Solesio M.E., Demirkhanyan L., Zakharian E., Pavlov E.V. (2016). Contribution of inorganic polyphosphate towards regulation of mitochondrial free calcium. Biochim. Biophys. Acta.

[6] Seidlmayer L.K., Juettner V.V., Kettlewell S., Pavlov E.V., Blatter L.A., Dedkova E.N. (2015). Distinct mPTP activation mechanisms in ischaemia-reperfusion: Contributions of Ca2+, ROS, pH, and inorganic polyphosphate. Cardiovasc. Res..

[7] Seidlmayer L.K., Gomez-Garcia M.R., Shiba T., Porter G.A., Pavlov E.V., Bers D.M., Dedkova E.N. (2019). Dual role of inorganic polyphosphate in cardiac myocytes: The importance of polyP chain length for energy metabolism and mPTP activation. Arch. Biochem. Biophys..

[8] Suess P.M., Watson J., Chen W., Gomer R.H. (2017). Extracellular polyphosphate signals through Ras and Akt to prime Dictyostelium discoideum cells for development. J. Cell Sci..

[9] Müller W.E., Wang S., Neufurth M., Kokkinopoulou M., Feng Q., Schröder H.C., Wang X. (2017). Polyphosphate as a donor of high-energy phosphate for the synthesis of ADP and ATP. J. Cell Sci..

[10] Müller W.E., Wang S., Ackermann M., Neufurth M., Steffen R., Mecja E., Muñoz-Espí R., Feng Q., Schröder H.C., Wang X. (2017). Rebalancing beta-Amyloid-Induced Decrease of ATP Level by Amorphous Nano/Micro Polyphosphate: Suppression of the Neurotoxic Effect of Amyloid beta-Protein Fragment 25–35. Int. J. Mol. Sci..

[11] Freimoser F.M., Hurlimann H.C., Jakob C.A., Werner T.P., Amrhein N. (2006). Systematic screening of polyphosphate (poly P) levels in yeast mutant cells reveals strong interdependence with primary metabolism. Genome Biol..

[12] Abramov A.Y., Fraley C., Diao C.T., Winkfein R., Colicos M.A., Duchen M.R., French R.J., Pavlov E. (2007). Targeted polyphosphatase expression alters mitochondrial metabolism and inhibits calcium-dependent cell death. Proc. Natl. Acad. Sci. USA.

[13] Kornberg S.R. (1957). Adenosine triphosphate synthesis from polyphosphate by an enzyme from Escherichia coli. Biochim. Biophys. Acta.

[14] Solesio M.E., Elustondo P.A., Zakharian E., Pavlov E.V. (2016). Inorganic polyphosphate (polyP) as an activator and structural component of the mitochondrial permeability transition pore. Biochem. Soc. Trans..

[15] Solesio M.E., Xie L., McIntyre B., Ellenberger M., Mitaishvili E., Bhadra-Lobo S., Bettcher L.F., Bazil J.N., Raftery D., Jakob U. (2021). Depletion of mitochondrial inorganic polyphosphate (polyP) in mammalian cells causes metabolic shift from oxidative phosphorylation to glycolysis. Biochem. J..

[16] Guitart-Mampel M., Urquiza P., Carnevale Neto F., Anderson J.R., Hambardikar V., Scoma E.R., Merrihew G.E., Wang L., MacCoss M.J., Raftery D. (2022). Mitochondrial Inorganic Polyphosphate (polyP) Is a Potent Regulator of Mammalian Bioenergetics in SH-SY5Y Cells: A Proteomics and Metabolomics Study. Front. Cell Dev. Biol..

[17] Hambardikar V., Guitart-Mampel M., Scoma E.R., Urquiza P., Nagana G.G.A., Raftery D., Collins J.A., Solesio M.E. (2022). Enzymatic Depletion of Mitochondrial Inorganic Polyphosphate (polyP) Increases the Generation of Reactive Oxygen Species (ROS) and the Activity of the Pentose Phosphate Pathway (PPP) in Mammalian Cells. Antioxidants.

[18] McCormack J.G., Halestrap A.P., Denton R.M. (1990). Role of calcium ions in regulation of mammalian intramitochondrial metabolism. Physiol. Rev..

[19] Borden E.A., Furey M., Gattone N.J., Hambardikar V.D., Liang X.H., Scoma E.R., Abou Samra A., LR D.G., Dennis D.J., Fricker D. (2021). Is there a link between inorganic polyphosphate (polyP), mitochondria, and neurodegeneration?. Pharmacol. Res..

[20] Kumble K.D., Kornberg A. (1995). Inorganic polyphosphate in mammalian cells and tissues. J. Biol. Chem..

[21] Pavlov E., Aschar-Sobbi R., Campanella M., Turner R.J., Gomez-Garcia M.R., Abramov A.Y. (2010). Inorganic polyphosphate and energy metabolism in mammalian cells. J. Biol. Chem..

[22] Bayev A.Y., Angelova P.R., Abramov A.Y. (2020). Inorganic polyphosphate is produced and hydrolysed in F0F1-ATP synthase of mammalian mitochondria. Biochem. J..

[23] Napolitano M.J., Nagele R.G., Shain D.H. (2004). The ice worm, Mesenchytraeus solifugus, elevates adenylate levels at low physiological temperature. Comp. Biochem. Physiol. A Mol. Integr. Physiol..

[24] Napolitano M.J., Shain D.H. (2005). Quantitating adenylate nucleotides in diverse organisms. J. Biochem. Biophys. Methods.

[25] Lang S.A., Shain D.H. (2018). Atypical Evolution of the F1Fo Adenosine Triphosphate Synthase Regulatory ATP6 subunit in Glacier Ice Worms (Annelida: Clitellata: Mesenchytraeus). Evol. Bioinform. Online.

[26] Hotaling S., Shain D.H., Lang S.A., Bagley R.K., Tronstad L.M., Weisrock D.W., Kelley J.L. (2019). Long-distance dispersal, ice sheet dynamics and mountaintop isolation underlie the genetic structure of glacier ice worms. Proc. Biol. Sci..

[27] Folmer O., Black M., Hoeh W., Lutz R., Vrijenhoek R. (1994). DNA primers for amplification of mitochondrial cytochrome c oxidase subunit I from diverse metazoan invertebrates. Mol. Mar. Biol. Biotechnol..

[28] Solesio M.E., Peixoto P.M., Debure L., Madamba S.M., de Leon M.J., Wisniewski T., Pavlov E.V., Fossati S. (2018). Carbonic anhydrase inhibition selectively prevents amyloid beta neurovascular mitochondrial toxicity. Aging Cell.

[29] Baltanas A., Solesio M.E., Zalba G., Galindo M.F., Fortuno A., Jordan J. (2013). The senescence-accelerated mouse prone-8 (SAM-P8) oxidative stress is associated with upregulation of renal NADPH oxidase system. J. Physiol. Biochem..

[30] Aschar-Sobbi R., Abramov A.Y., Diao C., Kargacin M.E., Kargacin G.J., French R.J., Pavlov E. (2008). High sensitivity, quantitative measurements of polyphosphate using a new DAPI-based approach. J. Fluoresc..

[31] Wang W., Nema S., Teagarden D. (2010). Protein aggregation--pathways and influencing factors. Int. J. Pharm..

[32] Kim K.S., Rao N.N., Fraley C.D., Kornberg A. (2002). Inorganic polyphosphate is essential for long-term survival and virulence factors in *Shigella* and *Salmonella* spp. Proc. Natl. Acad. Sci. USA.

[33] Rashid M.H., Kornberg A. (2000). Inorganic polyphosphate is needed for swimming, swarming, and twitching motilities of *Pseudomonas aeruginosa*. Proc. Natl. Acad. Sci. USA.

[34] Picard M., McEwen B.S. (2018). Psychological Stress and Mitochondria: A Conceptual Framework. Psychosom. Med..

[35] Gray M.J., Wholey W.Y., Wagner N.O., Cremers C.M., Mueller-Schickert A., Hock N.T., Krieger A.G., Smith E.M., Bender R.A., Bardwell J.C. (2014). Polyphosphate is a primordial chaperone. Mol. Cell.

[36] Kampinga H.H. (2014). Chaperoned by prebiotic inorganic polyphosphate molecules: An ancient transcription-independent mechanism to restore protein homeostasis. Mol. Cell.

[37] Cremers C.M., Knoefler D., Gates S., Martin N., Dahl J.U., Lempart J., Xie L., Chapman M.R., Galvan V., Southworth D.R. (2016). Polyphosphate: A Conserved Modifier of Amyloidogenic Processes. Mol. Cell.

[38] Beissinger M., Buchner J. (1998). How chaperones fold proteins. Biol. Chem..

[39] McIntyre B., Solesio M.E. (2021). Mitochondrial inorganic polyphosphate (polyP): The missing link of mammalian bioenergetics. Neural Regen. Res..

[40] Ellington W.R. (2001). Evolution and physiological roles of phosphagen systems. Annu. Rev. Physiol..

[41] Dahl J.U., Gray M.J., Jakob U. (2015). Protein quality control under oxidative stress conditions. J. Mol. Biol..

[42] Xie L., Jakob U. (2019). Inorganic polyphosphate, a multifunctional polyanionic protein scaffold. J. Biol. Chem..

